# Exploring the impact of role-playing exercises on cognitive and emotional processes: a social- and educational psychological perspective

**DOI:** 10.3389/fpsyg.2025.1645213

**Published:** 2025-09-15

**Authors:** Pär Löfstrand, Ingrid Zakrisson

**Affiliations:** Department of Psychology and Social Work, Faculty of Human Sciences, Mid Sweden University, Östersund, Sweden

**Keywords:** role playing activities, cognitive insight, emotional insight, mixed-method approach, educational psychology, social psychology

## Abstract

This study investigates the educational potential of role-playing exercises in addressing complex social phenomena such as attitude change, stereotyping, conformity, and racism. Building on prior research demonstrating the efficacy of role-play in reducing prejudice, this study aims to explore how such activities influence both cognitive and emotional engagement with these issues. The methodology included observation and evaluation of a professionally facilitated role-playing activity simulating the experience of being a refugee, conducted at an outdoor museum. Follow-up focus group interviews were conducted 2–3 years after the participants, students from local secondary schools engaged in the exercise. The findings indicate that the role-play had a lasting impact on students’ perceptions of forced migration and asylum seeking. Participants reported both cognitive insights and emotional responses, including empathy and personal reflection, as well as moments of detachment. The role-playing event was consistently described as one of the most memorable educational activities during their school years. A key implication from the evaluation and interviews is that, for role-playing to be an effective pedagogical tool in addressing sensitive topics such as xenophobia and prejudice, careful planning and structured facilitation are crucial to ensure meaningful and constructive outcomes.

## 1 Introduction

Factors such as stereotypes, conformity, attitudes and the upbringing of racism are important topics in social psychological courses. However, most often the literature is built up as brick stones with information and definitions of these topics. Education in the field could be enhanced by incorporating more activity-based methods, in which participants engage directly and interactively with the subject matter.

Role-playing has been widely recognized as an effective method for reducing prejudice, particularly among children, though it is also applicable to adults even if previous research indicates that it has stronger effects on children and adolescents (Tsergas et al., 2021). A notable example of this approach is Jane Elliott’s “Blue Eyes – Brown Eyes” exercise, a seminal natural experiment from the 1960s (Aboud and Levy, 2000; Pedersen et al., 2005). In this experiment, Elliott divided a classroom into two groups: one designated as “blue eyes” and the other as “brown eyes,” irrespective of the participants’ actual eye color. One of these groups was then subjected to discriminatory treatment, for example when she told the children that brown eyes were superior to blue, it fostered a sense of superiority and led to arrogant behavior among those placed in the favored group. From a cognitive dissonance (CD) perspective, this scenario can be understood as inducing dissonance in individuals who are prompted to engage in behaviors that conflict with their pre-existing attitudes. This as CD arises when there is an inconsistency between a person’s attitudes and their behavior. For example, if someone holds an attitude that promotes fairness and equality but then behaves in a discriminatory manner, this creates psychological discomfort, dissonance, to reduce this discomfort, individuals may either change their behavior to align with their attitude or adjust their attitude to justify the behavior (Festinger and Carlsmith, 1959). It has been suggested that newer cognitions are often more robust because they are more closely linked to behavior (Wicklund and Brehm, 2004). However, some scholars contend that reducing cognitive dissonance alone cannot fully account for the observed attitude changes. In real-world contexts, attitude shifts may be more accurately explained by internalization processes. For instance, after participating in a social experiment, individuals may come to realize that their previous biases were unfounded, leading to a genuine shift in their attitudes (Wicklund and Brehm, 2004). Another theoretical perspective, particularly relevant to children and adolescents, posits that prejudice originates from an egocentric viewpoint (Aboud and Levy, 2000). When students adopt the role of an out-group member, they are prompted to view the world from a perspective different from their own. This process has the ambition to cultivate empathy and enables them to better understand the emotional suffering of others.

Despite its widespread use as a tool for reducing prejudice and improving intergroup relations, there is a notable lack of follow-up studies assessing the long-term effectiveness of role-playing (Breckheimer and Nelson, 1976; Weiner and Wright, 1973). Much of what is known about the “Blue Eyes – Brown Eyes” experiment comes primarily from television documentaries, such as “The Eye of the Storm” by Peters (1970). In one of the few follow-up studies, adult participants reflected positively on their experiences, considering them both meaningful and impactful (Pedersen et al., 2005). Another experiment, based on the same scenario and conducted with teacher education students by Byrnes and Kiger (1990), found that most participants viewed the experience as significant, with a moderate reduction in prejudice. However, many participants also reported experiencing stress during the simulation. Several studies involving children and adolescents have found positive, though weak, effects (Aboud and Levy, 2000). A comprehensive meta-analysis including results from 26 studies on prejudice reduction by McGregor (1993), which draws statistically generalizable conclusions revealed that role-playing significantly reduced racial prejudice. However, it was no more effective than other forms of anti-racist education. Additionally, role-playing appeared to reduce prejudice more effectively among children and adolescents compared to adult participants.

Evaluations of the long-term effects of role-playing, or other pedagogical approaches such as reading about racist abuse or watching movies concerning the topic, aimed at raising awareness of racism, remain limited (Adams et al., 2008; Kernahan and Davis, 2010). One of the few studies in his area was conducted in Australia and focused on adults’ attitudes toward Aboriginal people. The study incorporated role-playing as witnesses to a racist incident, among other measures (Hill and Augoustinos, 2001). While participants reported reduced prejudice immediately after the event, these effects were short-lived, with prejudice levels returning to their original state 3 months later. There are several possible explanations for these short-lived effects. One is that participants may display reduced prejudice immediately after the event due to a desire to conform to perceived social expectations, rather than because of a genuine change in underlying beliefs. Alternatively, ongoing social pressures or exposure to prejudiced environments after the intervention may gradually erode any initial attitude change. Additionally, methodological limitations may contribute to this outcome, as controlling for external variables in real-world settings is inherently challenging, making it difficult to isolate the true effect of the intervention (Paluck et al., 2021; Hsieh et al., 2022).

A Norwegian study, involving children and adolescents examined the effects on a jigsaw cooperative learning experience rather than role-playing. This technique involves participants becoming experts on a specific portion of a topic and then teaching it to others, with the goal of fostering interdependence and cooperation. While the study found no long-term effects, though a few minor positive effects were observed in the short term (Bratt, 2008). Another more recent study explored how perspective taking could reduce prejudice, their results indicate that individuals with stronger perspective taking are more affected than those with weak perspective taking (Szekeres et al., 2024). Some evaluations of racial awareness education have shown that while the immediate impact on racism may diminish over time, emotional insights and interethnic interactions tend to increase, (Kernahan and Davis, 2010), particularly when methods emphasizing the perspectives of marginalized groups, such as for example immigrants or asylum seekers are employed (Adams et al., 2008). These findings highlight the need for further research on the long-term effects of such interventions. Several factors may explain the difficulty in documenting lasting positive effects of role-playing on prejudice. One key issue is the methodological approach; evaluations are often not conducted by trained researchers who can design rigorous natural experiments (Aboud and Levy, 2000). Additionally, there may be issues with the variables measured. For example, attitudes that are too broad may fail to align closely with the specific behaviors enacted during the role play. Research on attitude-behavior consistency suggests that the more specific the attitude, the greater the correspondence with the target behavior, specifically, when an individual’s attitude is directed toward a clearly defined behavior rather than a general category. (Davidson and Jaccard, 1979; Kraus, 1995; Tsergas et al., 2021).

There is also reason to expect people to differ in their proneness to attitude change. For example, as mentioned above, younger individuals seem more susceptible than adults. Gender may also play a part, as prejudice often displays gender differences (Akrami et al., 2000; Navarrete et al., 2010; Sidanius and Pratto, 1999), at least if explicit prejudice is concerned (Ekehammar et al., 2003). Women most often display lower levels of prejudice to any target group than men. The reason for this may be manifold. Explicitly prejudiced and non-prejudiced people have been found to be equally aware of cultural stereotypes (Akrami and Ekehammar, 2005; Devine, 1989) and women have in fact been shown to display higher levels of implicit prejudice than men, although they score lower on explicit measures (Ekehammar et al., 2003). This apparent discrepancy raises important methodological and theoretical questions about the relationship between implicit and explicit prejudice. Research suggests that the correlation between implicit and explicit measures is typically modest, partly because they tap into different cognitive processes: implicit attitudes are automatic and unconscious, whereas explicit attitudes are deliberate and controlled (Greenwald et al., 2009). It has further been shown than the association between explicit and implicit attitudes are dependent on the level of commitment to control prejudiced reactions (Akrami and Ekehammar, 2005), suggesting that women are more committed to inhibit socially undesirable overt responses in this realm than men. Perhaps then there are differences in commitment between men and women to reduce their levels of prejudice. Research, both from terror management perspectives (Arndt et al., 2002), as well as the outgroup male target hypothesis (Sidanius and Veniegas, 2000) points to that certain social cognitions, for example intergroup bias, is related to different psychological systems for men and women, especially in relation to various forms of threat; such as collective constructs among men and relational constructs among women (Arndt et al., 2002) and dominance motives for men and fear of sexual coercion for women (Navarrete et al., 2010). These underlying motives, such as dominance striving in men may under certain conditions contribute to resistance to prejudice reduction efforts, while in other circumstances facilitate prejudice reduction, and maybe these processes are different for men and women. One of the proposed dynamics behind role playing is that individuals is encouraged to play the role of an out-group member, they learn to adopt a perspective different from their own, and to understand the painful feelings of others, through the process of empathy (Aboud and Levy, 2000). This not only suggest that empathy is an important process mediating the effect of role playing, in fact it implies that the levels of empathy increase during a role-playing experience. Studies have shown that this is possible through training programs, for example the jigsaw classroom or conflict resolution workshops where the participants is trained to hold the other sides perspective (Colca et al., 1982; Stephan and Finlay, 1999). Moreover, consistent differences between men and women have previously been found, with women scoring higher on empathy than men (Auyeung et al., 2009; Garaigordobil et al., 2009; Mehrabian and Epstein, 1972; Reniers et al., 2011). Furthermore, empathy is often found to be related to various forms of prejudice, and to account for gender differences in prejudice (Bäckström and Björklund, 2007; Pettigrew and Tropp, 2008). According to Pettigrew and Tropp (2008) is empathy defined as the ability to take the perspective of others and feel concern for their well-being. Moreover, according to them empathy plays a key role in reducing prejudice by fostering more positive attitudes. There are few studies reporting gender differences in susceptibility to role playing. One of the few had teenagers role play an intervention concerning the consequences of teen pregnancy (Saltz et al., 1994). The main finding was that girls responded more favorably to the intervention than boys. As an unwanted pregnancy presumably has more serious consequences for a girl than for a boy, it is reasonable to believe the female participants were more committed to change their attitudes in this respect. This indicates that men and women may be differently committed to change their attitudes, and under different circumstances. Why such studies are scarce is hard to tell, there are, however, theoretical reasons that gender differences are likely. It has been suggested that gender differences are due to the kind of appeal made (see Briñol and Petty, 2005); the gender difference is reduced when the appeal is based on reciprocity rather than sympathy. This, again, suggest that men and women are differently motivated to attitude change, under certain conditions.

This study aims to explore the long-term effectiveness of role-playing exercises in reducing prejudice, with a particular focus on how cognitive and emotional processes influence attitudes and stereotypes. The study seeks to examine how role-playing can foster empathy, alter perspectives, and potentially change prejudicial beliefs by inducing cognitive dissonance and encouraging individuals to adopt the viewpoint of an out-group member. Furthermore, the research aims to investigate how gender, empathy, and motivation may shape individuals’ susceptibility to attitude change, considering previous findings of gender differences in empathy and prejudice reduction. The study will also address the lack of follow-up research on the long-term effects of role-playing exercises in educational settings and the challenges associated with evaluating these interventions.

## 2 Methodology

### 2.1 Participants

A total of 137 students (comprising 70 females and 67males, age 16–19) participated in a role-playing exercise held at an outdoor museum. Additionally, eight students (17–19 years) participated in two follow up focus group interviews, one consisting of four women and the other of four men. The focus group participants were recruited by their teacher during a lesson in civics and volunteered to take part as participants.

### 2.2 Role playing–taking the perspective of a refugee

The exercise was organized and conducted at an outdoor museum in Sweden, with the activity designed by the museum’s pedagogical staff. As researchers, we were actively involved in the process from a research perspective, collecting data through observations, interviews, and surveys at three key intervals: 3 weeks before the exercise, immediately after, and 3 weeks following the exercise.

#### 2.2.1 Setup for the role play

The role-playing activity took place at the outdoor museum, with participants gathering in a rehearsal hall at 8:15 a.m. Upon arrival, they were informed about the purpose of the activity and the basic principles of role-playing. They were also made aware that their participation was voluntary and that they could withdraw at any time. Additionally, participants were reassured that professional staff were available throughout the day to provide support or to speak with anyone who felt uncomfortable or wanted to discuss their experience.

The session began with a screening of a documentary film aimed at shedding light on the refugee experience. The film presented information on the challenging conditions refugees face, ongoing conflicts, and current refugee statistics. It also included interviews with individuals residing in refugee camps, as well as those who had fled war zones, sharing personal stories of their journeys to safety in a new country.

At the end of the film, participants were handed an envelope containing a letter with detailed information about a fictional individual currently living as a refugee. The letter included personal details such as the person’s name, age, gender, religion, and ethnicity, as well as the reasons behind their refugee status. The letter also described the individual’s relationships with other refugees in the room, who were role-played by other participants. Additionally, each participant was given a personal belonging — such as a photograph, teddy bear, train ticket, money, or an ID card—along with an explanation of its significance to the character. After approximately 30 min of preparation, participants were asked if they were ready to fully embody their character for the role-play.

Once everyone was ready, they were introduced to their co-role players, who were assigned to act as their relatives. Suddenly, the room went dark, and participants were informed that they were in a room waiting for strangers who were supposed to help them escape to a neighboring country. After approximately 10 min, two actors dressed as refugee smugglers entered the room, appearing stressed and anxious. They began to divide the larger group into smaller subgroups of four to five participants. This marked the first role-playing action, as participants were potentially separated from those assigned to be their relatives.

In the next phase, each subgroup was led out of the building, and from there, different scenarios unfolded for each group.

For example, one group was forced into an old minivan, while others were instructed to silently walk through a forest to avoid detection by border control. After a period of travel, the groups arrived at an old building and were told to keep the lights off and stay vigilant to avoid being discovered by others. Soon after, a police car with actors dressed as police officers appeared in front of the building. At this point, participants had to quickly find places to hide. The police officers entered the building, and once all the refugees (participants) were found, they were escorted to another building where they were instructed to wait.

In this new building, participants encountered actors speaking a foreign language they could not understand. They were forced to communicate using gestures or drawings. This portion of the role-play was the longest of the day, intended to simulate the long waiting periods refugees often experience. After some time, participants were individually introduced to a staff member playing the role of a refugee coordinator. With the help of an actor acting as an interpreter, they were asked to provide personal information and explain why they were seeking asylum.

Once all participants had completed this step, the role-playing activity was concluded, and everyone reconvened in the rehearsal hall. There, the pedagogical staff facilitated a debriefing session, where participants had the opportunity to share their experiences, reflections, and thoughts on the day’s events.

## 3 Results

After participating in the role play, the participants were asked to write down their own reflections on the activity. A total of 193 statements related to emotional or cognitive factors were found (Table 1). The emotional factors were divided to three sub-categories, emotional insight, negative emotions, sympathy, and denial. The cognitive factors consisted of cognitive insight, egocentrism, and cognitive dissociation. The most frequent comments were categorized as negative emotions and egocentric thoughts. There was a significant difference according to gender for three of the seven categories. The girls exhibited significantly more negative emotions, χ^2^(1, *N* = 137) = 21.61, *p* < 0.001; greater cognitive insight, χ^2^(1, *N* = 137) = 8.38, *p* < 0.005; and higher emotional insight, χ^2^(1, *N* = 137) = 12.03, *p* < 0.002. There were no significant differences between groups in the other four categories: egocentrism, χ^2^(1, *N* = 137) = 1.98, *p* > 0.20; cognitive dissociation, χ^2^(1, *N* = 137) = 2.55, *p* > 0.10; sympathy, χ^2^(1, *N* = 137) = 1.22, *p* > 0.26; and denial of emotions, χ^2^(1, *N* = 137) = 0.12, *p* > 0.70.

**Table 1:** Participants’ reflections on the role-play: emotional and cognitive insights.

Gender	Emotional aspects			Cognitive aspects			
	Emotional insight (15)	Negative emotions (42)	Sympathy (7)	Denial of emotions (19)	Cognitive insight (38)	Egocentrism (43)	Cognitive dissociation (29)
Girls	“Was it for real? And when I thought of that I was scared”	“I felt stressed, and I was nervous”	“I feel sorry for *them;* I believe that we help too few to come to our country”	“To understand, I had to turn my emotions off”	“I had to remind myself that it wasn’t for real and only a part in a role play”	“That I (my character) did not get asylum”	“It was no seat belts in the buss.”
Boys	“It was good to learn a role and to gain under-standing about how hard it can be to come to a foreign country”	“It felt strange”	“More sympathy for refugees”	“It was only a role play; I had no feelings at all”	“I got some insight in how it is to be a refugee”	“I did not *feel* anything except pain in my ears when she who sat in the car screamed so loud”	“The Eurodac instrument did not look trustworthy”

### 3.1 Focus group interviews

Two focus group interviews were conducted for the study. One group comprised four female students (pseudonyms: Anna, Caroline, Ida, and Felicia), while the other included four male students (pseudonyms: Marcus, Peter, Sam, and Tomas). All participants were classmates enrolled in the same academic program at a secondary school at the time of the interviews. Given that participation in a role-playing event is common among students during their 9th-grade year, it was anticipated that several group members would have prior experience with the activity. This expectation was confirmed, as all participants reported having taken part in the role-playing exercise at the local open-air museum 3 years earlier. Both focus group interviews employed a semi-structured format (Adeoye-Olatunde and Olenik, 2021), guided by four central questions designed to elicit open and reflective responses. The discussions were audio-recorded using an Olympus digital voice recorder.

#### 3.1.1 Focus group discussion–girls

In response to the first question concerning their experiences with school-based education on racism and xenophobia, participants generally agreed that while these topics were perceived as important, they were not systematically integrated into the formal curriculum. Anna remarked, “It is really important that school address these kinds of questions; most often we discussed it, but not so much during the lessons,” suggesting that conversations about racism and xenophobia tended to occur informally—often outside of structured classroom settings, such as during breaks. Ida pointed to the social difficulties of confronting these issues, stating, “Some are really racist, and it can be tough to speak out against them,” indicating that peer dynamics could act as a barrier to open discussion. Anna further clarified that when such topics were addressed in class, they were typically confined to specific subjects, most notably civics: “We mostly talked about these issues during civics, especially when we discussed World War II, like concentration camps.”

When asked how schools should address issues of racism and xenophobia, the female participants promptly highlighted the value of the role-playing event they had participated in during high school. Felicia reflected on the experience, stating: “My class was at Jamtli (the museum) once and took part in a thing where we role-played refugees for a day. That was good, as we talked about how we should treat each other. We also discussed how we may help people who have it difficult when we came back to school.” Both Anna and Ida confirmed their involvement in the same event. Anna remarked, “My class was also there, it was really great,” while Ida added, “Yes, now I remember we took part. There should be more of these kinds of activities so that everyone can participate.” These reflections indicate that experiential learning activities—such as the refugee role-play—were regarded as meaningful and effective pedagogical tools for fostering empathy and encouraging dialogue about racism, xenophobia, and social responsibility.

In response to the third question, which focused on their high school experiences related to immigration and refugee issues, the discussion once again returned to the previously mentioned role-playing activity. Anna opened the conversation by noting, “We already talked about the role-play at the museum,” indicating the enduring impact of the experience. Felicia observed that classroom discussions on immigration were relatively limited and often framed in binary terms: “We did not talk so much about it during lessons. If we did, it was almost always ‘pro’ or ‘con.’” Caroline added, “Yes, some of the boys were really against immigrants,” suggesting that peer attitudes also shaped the classroom climate. Felicia went on to reflect on the role of teachers, stating, “I think that not all the teachers had the guts to talk about immigration because they did not know how to handle it. We had one teacher in civics who talked about it, and that was good.” She reiterated the effectiveness of the role-playing experience, emphasizing the importance of external facilitators: “The day at the outdoor museum was really good because we did not know the people who worked there, and they were good—it made us listen.” These accounts highlight both the challenges educators may face when addressing sensitive topics and the potential of experiential learning environments to foster meaningful engagement.

In response to the final question—how schools should approach teaching about immigration and the experience of being a refugee—participants expressed views consistent with earlier parts of the discussion. Felicia once again highlighted the value of the outdoor museum activity, stating, “The day at the outdoor museum was good.” Caroline concurred, emphasizing its educational potential: “Yes, everyone should take part to learn more facts and to understand better. But not everyone wants to—they have already decided what they believe, and they are just angry at immigrants.” These reflections suggest that, while formal instruction on immigration was perceived as either limited or polarized, experiential learning opportunities were regarded as effective in promoting empathy, deepening understanding, and engaging students with complex and often sensitive social issues.

#### 3.1.2 Focus group discussion–boys

Responses to the first question, which explored students’ experiences with high school education on racism and xenophobia, revealed a perceived lack of depth and consistency in how these topics were addressed. Marcus remarked that “there was too little” focus on racism and xenophobia, emphasizing the need for more sustained classroom dialogue: “There should have been more discussions.” Peter recalled a specific educational moment, stating, “We saw a movie one time—I do not remember the name—but it was about some neo-Nazis in the USA, which was nice.” These reflections suggest that, although some content related to racism and xenophobia was included in their education, opportunities for meaningful engagement and critical discussion were perceived as limited.

In response to the question of how schools should address issues of racism and xenophobia, the boys did not reference the earlier role-playing activity but instead proposed alternative educational strategies. Peter suggested that hearing directly from individuals with lived experiences would be impactful: “It would have been meaningful if someone with personal experiences could visit the school. A friend of mine had a visit from an elderly man who had been in a concentration camp during the Second World War.” Tomas confirmed the effectiveness of such encounters, adding, “We had that kind of visit—everyone was really affected, and some of the girls cried.” These reflections underscore the boys’ preference for firsthand testimonies and emotionally resonant experiences as powerful tools for fostering awareness and empathy around issues of racism and xenophobia.

In response to the third question, which focused on their experiences with high school education on immigration and refugee issues, the boys recalled participating in the role-playing activity at the outdoor museum. Marcus described the event as “funny,” while Peter echoed the sentiment, humorously noting, “We did not get any food—I was really hungry.” Sam also remembered the activity, stating, “We were at the outdoor museum, I think it was in 9th grade. There should be more of these activities.” Tomas redirected the discussion to a different educational experience, recalling a school visit from a man who had been imprisoned in a concentration camp during the Second World War: “After his visit, we talked about these issues during civics.” Collectively, the boys expressed a desire for more open discussion in school settings. Peter remarked, “I think the youth recreation leaders were good; it’s more natural to talk with them than in the classroom. I do not know if they are fully qualified, but at least we were allowed to talk.” These responses suggest a clear preference for interactive, relatable, and emotionally engaging learning formats—both within and beyond the formal classroom—as a means of addressing complex and sensitive social issues.

In summary, both the female and male participants identified a range of educational experiences—such as films, guest speakers, and the role-playing activity—as meaningful approaches to addressing racism. They noted that discussions on these topics often occurred more frequently outside of formal classroom settings, such as during breaks or with non-teaching staff. While all participants recalled engaging with issues related to racism and xenophobia at school, the nature and extent of these discussions varied depending on the context, including differences in school environment, teachers, and other personnel. The role-playing event, which took place 3 years earlier, was clearly memorable; participants described it using terms such as “good,” “funny,” and “important,” suggesting that it left a lasting impression.

## 4 Discussion

The findings of this study suggest that simulations and role-playing exercises can have a significant and immediate impact on students’ cognitive and emotional responses to complex social issues such as stereotyping, prejudice, and asylum seeking. The focus group interviews, conducted 2–3 years after the role-playing activity, indicate that the experience left a lasting impression on the eight participants. The interviewed participants came from different schools and attended the role-play at different times, which suggests that their experiences were meaningful. This consistency makes it reasonable to consider whether the positive outcomes could be generalized. This long-term retention highlights the potential of experiential learning to enhance deep engagement and reflection. Moreover, the study reveals that the social and educational context plays a critical role in shaping how stereotypes are formed, discussed, and either reinforced or challenged. Thematic analysis of the interview data showed that the role-play stimulated not only empathy and cognitive awareness but also elicited a range of emotional responses—including denial, discomfort, and egocentric perspectives. These varied reactions underscore the complexity of affective learning and point to the importance of guided reflection and facilitation when using role-play to address sensitive and potentially polarizing topics.

Another notable finding was the gender differences in how participants experienced and reflected on the role-playing activity. Chi-square tests revealed that female participants reported a higher frequency of negative emotional responses. Along with more nuanced emotional and cognitive reflections. This aligns with previous research suggesting that women tend to exhibit lower levels of implicit bias and greater empathetic sensitivity (e.g., Ekehammar et al., 2003). Thematic analysis indicated that both negative emotional reactions such as discomfort and sadness and cognitive mechanisms such as cognitive insight and cognitive dissociation were employed by participants when evaluating their experiences, with two primary themes emerging: negative emotional responses and cognitive insights.

The prevalence of negative emotions—such as discomfort, sadness, or empathy—suggests that the activity resonated on an affective level, challenging participants to emotionally engage with the challenges faced by refugees. Simultaneously, the presence of cognitive insights—such as reflections on social injustice, stereotyping, or the mechanics of asylum seeking—indicates that the exercise also helped deeper intellectual understanding. These dual outcomes point to the potential of role-playing as a tool for promoting both emotional engagement and critical reflection on complex social issues.

The focus group interviews further demonstrated that the impact of the role-playing activity was enduring. Even 2–3 years after the event, participants were able to recall and describe the experience with considerable detail, underscoring its lasting impression. Notably, gender differences were again apparent in these retrospective accounts. Female participants tended to retain more vivid and emotionally charged memories of the activity and continued to express both cognitive and emotional engagement. This suggests a deeper and more sustained internalization of the experience among the female students, highlighting the potential for gendered patterns in how experiential learning is processed and remembered over time.

These findings underscore the importance of considering gender dynamics in the design and evaluation of this kind of learning interventions. They also reinforce the potential of role-play as a method that, when carefully facilitated, can generate both immediate and enduring benefits across emotional and cognitive domains.

This study was based on a small sample and focused on a single, professionally facilitated role-playing exercise. As such, the findings may not be directly generalizable to classroom-based implementations of similar activities. The long-term effects reported by participants were likely influenced by the fact that the activity took place outside the regular school environment, breaking the routine and thereby becoming more memorable than a typical classroom lesson. While this may be viewed as a limitation in terms of ecological validity, it also suggests that conducting such activities in alternative settings may enhance their impact and retention. Another limitation of the study is the absence of explicit quantitative measures, such as the Modern Racism Scale (McConahay, 1986) or the Social Dominance Orientation Scale (Pratto et al., 1994). The inclusion of such instruments in future research could provide more robust data on attitudinal changes by enabling pre- and post-intervention comparisons. In the present study, these measures were excluded due to the methodological challenges associated with isolating the effects of the intervention in a quasi-experimental design. Incorporating such tools would be more feasible under controlled experimental conditions. Another limitation of this study is the lack of consideration for participants’ individual experiences with asylum seeking. Future research could address this by including questions related to both personal experiences and knowledge of the asylum-seeking process.

A central question emerging from this study, and supported by prior research, is whether activity-based methods such as roleplaying should be more systematically integrated into educational practice, both at the secondary and tertiary levels. The findings of this study, in line with existing literature, suggest that experiential pedagogical approaches such as role-playing can significantly enhance both cognitive understanding and emotional engagement with complex social issues. However, the effectiveness of such methods appears to depend heavily on the quality, context, commitment among the participants and structure of implementation.

Naturally, we are all influenced by events and meetings with other people in our personal lives as well as by broader societal and global shifts. This presents a challenge for longitudinal research in experimental/quasi-experimental settings, as there are numerous uncontrolled (confounding) variables that may affect individuals and their attitudes over time. For example, in the context of this study, the asylum crisis following the war in Syria had a significant impact on many European countries, and public responses to it have been deeply divided. One perspective is to view the world as socially constructed. As Potter (1996, 2012) argues, every emotionally charged experience or new encounter shapes and reshapes our understanding of the world. A complementary interpretation is offered by Goffman (1949, 2023) who suggests that individuals adopt different *masks* in different social contexts, performing roles that align with those masks. The role-playing activity used in this study can be seen as temporarily providing participants with such a mask, that of an asylum seeker. This embodied experience may foster a more profound empathy and a deeper awareness of the challenges faced by asylum seekers. In turn, this heightened understanding could potentially shift participants’ perspectives on refugee-related issues and policies.

Future research should further explore the functional dimensions of empathy, motivation, and emotion in this context, clarifying how these constructs operate within specific interpersonal dynamics. This includes, for example, distinguishing between cognitive and affective empathy, exploring intrinsic versus extrinsic motivational drivers, and mapping specific emotional responses to role-playing interactions.

In this case, the involvement of professional actors and the location outside the traditional classroom setting contributed meaningfully to the depth and memorability of students’ experiences. These factors likely strengthened the impact of the activity, underlining the importance of context and facilitation in experiential learning. Based on these insights, we recommend that if role-playing is to be employed as a method for addressing sensitive topics such as prejudice, xenophobia, or attitude change, it should be preceded by thorough planning and structured integration into the broader curriculum. Moreover, especially when the method is being introduced for the first time, it is advisable to have at least two trained facilitators present. This ensures proper guidance, emotional support, and pedagogical effectiveness, while also allowing the facilitators to support one another throughout the activity.

Finally, we argue that higher education—particularly courses in fields such as social psychology—should more actively incorporate experiential learning methods, including role-play. Given their demonstrated potential to promote deep cognitive engagement and emotional understanding, such approaches offer valuable opportunities for students to explore complex social dynamics in a meaningful and hopefully lasting way. These methods could be of good use when for example introducing how stereotypes occur, to understand the prisoner’s dilemma or to get a better understanding on how attitudes might be changed.

## Data Availability

The raw data supporting the conclusions of this article will be made available by the authors, without undue reservation.
